# Search for *Cryptococcus neoformans/gattii* Complexes and Related Genera (*Filobasidium, Holtermanniella, Naganishia, Papiliotrema, Solicoccozyma, Vishniacozyma*) spp. Biotope: Two Years Surveillance of Wild Avian Fauna in Southern France

**DOI:** 10.3390/jof8030227

**Published:** 2022-02-24

**Authors:** Sébastien Bertout, Tiphany Gouveia, Donika Krasteva, Julie Pierru, Cyrille Pottier, Virginie Bellet, Emilie Arianiello, Florian Salipante, Frédéric Roger, Pascal Drakulovski

**Affiliations:** 1 Laboratoire de Parasitologie et Mycologie Médicale, UMI 233 TransVIHMI, University of Montpellier, IRD, INSERM U1175, 15 Avenue Charles Flahaut, 34093 Montpellier, France; sebastien.bertout@umontpellier.fr (S.B.); tiphany.gouveia@umontpellier.fr (T.G.); donika.krasteva@umontpellier.fr (D.K.); cyrille.pottier@umontpellier.fr (C.P.); virginie.bellet@umontpellier.fr (V.B.); frederic.roger1@umontpellier.fr (F.R.); 2 Centre Régional de Sauvegarde de la Faune Sauvage, LPO Hérault, 15 rue de Faucon Crécelerette, 34560 Villeveyrac, France; julie.pierru@lpo.fr (J.P.); emilie.arianiello@lpo.fr (E.A.); 3 Department of Biostatistics, Clinical Epidemiology, Public Health, and Innovation in Methodology, Nîmes University Hospital Center, University of Montpellier, 34000 Nîmes, France; florian.salipante@chu-nimes.fr

**Keywords:** biotope, birds, carriage, reservoir, tremellomycetes

## Abstract

Fungi belonging to the *Cryptococcus* genus and related genera (*Filobasidium*, *Holtermanniella*, *Naganishia*, *Papiliotrema*, *Solicoccozyma*, *Vishniacozyma*) are encapsulated yeasts found in either the environment or animal sources. However, the precise biotopes of most species remain poorly defined. To assess whether wild birds from southern France can carry or spread the most pathogenic species (i.e., species belonging to the *C. neoformans* and *C. gattii* complexes), as well as lesser-studied species (non-*neoformans/gattii Cryptococcus* and former *Cryptococcus* spp.), 669 birds belonging to 89 species received for care over a two-year period at the Centre de Protection de la Faune Sauvage of Villeveyrac (Bird Protection League nongovernmental organization (NGO) care center) were sampled. Samples were cultured, and *Cryptococcus* and former *Cryptococcus* yeasts were identified by PCR sequencing. The purpose was to evaluate whether there was any health risk to local populations or care personnel in aviaries and gather new data on the ecological niches of lesser-known species. One hundred and seven birds (16%) were found to be positive for at least one *Cryptococcus* or former *Cryptococcus* species. No yeasts belonging to the highly pathogenic *C. neoformans* or *C. gattii* complexes were isolated. However, diversity was notable, with 20 different Cryptococcus or former Cryptococcus species identified. Furthermore, most bird–yeast species associations found in this study have never been described before.

## 1. Introduction

*Cryptococcus* spp. are yeasts with a natural biotope that varies from species to species. The genus has recently undergone major classification changes, with the separation of *C. neoformans* and *C. gattii* species into two complexes of two (*C. neoformans/C. deneoformans*) and five (*C. gattii/C. bacilisporus/C. deuterogattii/C. tetragattii/C. decagattii*) species, respectively [1,2]. Species other than *C. neoformans/C. gattii* have also undergone taxonomic reclassifications and have been redistributed between the *Cryptococcus* genus and other genera, including *Cutaneotrichosporon*, *Cystofilobasidium*, *Filobasidium*, *Hanalea*, *Holtermanniella*, *Naganishia*, *Papiliotrema*, *Solicoccozyma*, *Vishniacozyma,* and *Vanrija*. For instance, *C. magnus*, *C. chernovii,* and *C. oeirensis* were reclassified as *Filobasidium magnum*, *F. chernovii,* and *F. oeirense*, respectively. *Cryptococcus festucosus* was reclassified as *Holtermanniella festucosa. Cryptococcus albidus*, *C. diffluens*, *C. saitoi*, *C. liquefasciens,* and *C. uzbekistanensis* were reclassified as *Naganishia albida*, *N. diffluens*, *N. globosa*, *N. liquefasciens,* and *N. uzbekistanensis* respectively. *Cryptococcus laurentii* and *C. terrestris* were reclassified in the *Papiliotrema* genus. *Cryptococcus aerius* and *C. carnescens* were renamed *Solicoccozyma aeria* and *Vishniacozyma* *carnescens*, respectively [3].

The members of the *C.* *neoformans/C. gattii* complexes, which are the main aetiological agents of cryptococcal meningitis, responsible for 181,000 deaths worldwide annually [4] are the most virulent species among those studied. They are followed by *N. albida* and *P. laurentii*, which account for 80% of non-*neoformans/gattii* cryptococcal infections. The remaining 20% of non-*neoformans/gattii* cryptococcal mycoses are due to 16 species of rare *Cryptococcus* and former *Cryptococcus* [5].

Given their medical importance, the control of potential reservoirs of these yeasts appears to be of utmost importance. Historically, *C. neoformans sensu lato* (*s.l.*) was first isolated from pigeon (*Columba livia*) droppings [6,7,8], suggesting this is a bird-borne fungus. However, isolations from other animal sources, particularly insects and bat guano, were subsequently reported [9,10,11,12]. *C. neoformans s.l.* isolates have also been retrieved from vegetal material, including trees and tree trunks [13,14] and vegetables/fruits [15,16]. Moreover, it was shown that this yeast is able to complete its whole life cycle, including mating, on plants [17,18], suggesting that its true reservoir could be vegetal. The same pattern has been observed for the *C. gattii* species complex, the other aetiological agent of cryptococcal meningitis. Initially, this complex was thought to be exclusively associated with trees, tree hollows, or decaying tree parts of eucalyptuses (*Eucalyptus camaldulensis*) [19,20]; however, *C. gattii s.l.* strains were subsequently isolated from additional tree species, such as *Pinus*, *Olea,* and *Ceratonia* [21,22,23]. While originally thought to be strictly plant-borne, *C. gattii s.l.* strains have also been found in bat guano [24], insect nests, and insect frass [25,26] bird excreta, [27,28] and in dens/burrows of at least one mammal, the African hyrax [29]. Isolates were also obtained from a wide range of animals, such as gray squirrels [30], porpoises [31], and pets such as dogs and cats, during the 1999 Vancouver outbreak. However, in this outbreak, the tested animals were not the natural reservoir for *C. gattii s.l*. The range of sources of rare *Cryptococcus* and former *Cryptococcus* species (hereafter abbreviated RCFC) is also wide. *Naganishia albida* and *P. laurentii* have been found in the environment in soil [32,33,34], dust [35], plants [36,37], and water [38] as well as bird droppings [39]. Some other species, such as *Filobasidium wieringae*, *F. oierense*, and *V. carnescens*, have been isolated from asymptomatic carrier mammals, such as feral cats [40]. Other species, such as *Naganishia adeliensis*, *N. diffluens*, *N. uzbekistanensis* and *P. terrestris*, were initially isolated from environmental sources [41,42,43] but later from animals. Finally, some species, such as *S. aeria*, are not considered animal-borne because they have been isolated from only the environment thus far [44].

The variety of ecological niches in which these various cryptococcal species can be found raises two issues. Concerning *C. neoformans* and *C. gattii* species complexes, the most virulent species, the assessment, management, and control of a potential reservoir is difficult while these species impose a heavy burden on health systems in countries with a high HIV prevalence [4]. Concerning the less virulent RCFC species, the absence of a clear biotope or reservoir hinders the understanding of their biology while some of them could be responsible for human or veterinary infections [5]. Given the strong association of C. *neoformans s.l.* and *C. gattii s.l.* with birds and bird excreta and the existence of the same indicators for rare *Cryptococcus* species, we decided to perform a study on avian fauna from Occitanie, southern France. The objective was to assess whether *C.* *neoformans/C.* *gattii s.l*. or RCFC are naturally present in the feces of animals. If so, they could eventually pose a risk to local human or wild/farm/pet animal populations, similar to other bird-borne gastrointestinal opportunists, such as *Chlamydia psittaci* and *Candida glabrata* [45]. Furthermore, most studies on the biotope/reservoir of animal-borne *Cryptococcus* species are performed in urban areas and in domestic animals or captured fauna (particularly in bird markets or zoos) because they search specifically the most clinically significant species; *C. neoformans/C. gattii s.l.* [46]. On the contrary, we decided to focus on wild fauna samples. This was done to avoid a possible bias related to sampling performed in animals that interact with humans (pets or captive animals) or in an anthropogenized environment (such as an urban area), in which the situation may be different from the wild.

## 2. Materials and Methods

### 2.1. Sampling Centre

Sampling was conducted at the Centre de Protection de la Faune Sauvage, Ligue de Protection des Oiseaux (LPO) Hérault (Centre for Wild Fauna Protection, Bird Protection Association, Hérault, France). This facility is located in Villeveyrac (34560 France, GPS: 43°28′47.6″ N 3°36′49.3″ E) and is the departmental center for the care of wild fauna in distress. It receives approximately 3000 animals per year on average from the Hérault, Aude, and Pyrénées Orientales areas (see Supplementary Data S1); the admitted animals are mostly wild birds (88% of animals), followed by mammals (11.7%) and a small number of reptiles (0.3%). Its primary purpose is to care for wild animals that are wounded or in distress and rehabilitate them for release back into the wild. It should be noted that the center does not care for pet animals or wild species that are considered domestic or invasive.

### 2.2. Animal Population Sampling

From May 2017 to July 2019, ten to twenty birds among the individuals who received daily care at the center were randomly chosen every 7 to 10 days for sampling. A total of 669 wild birds belonging to 71 genera and 89 species (+1 undetermined at the species level) were sampled.

### 2.3. Method of Sampling

During the study, each bird to be tested was kept in a separate cage during the care period. Cages were cleaned with sodium hypochlorite between each individual to avoid cross-contamination. The age, species, reason for admission, treatment, and swab method were documented for each individual. Sampling was performed by direct cloacal swabbing when possible. Alternatively, cage swabbing was performed when concerns about the well-being and compliance of animals or excessive risks to care personnel made direct cloacal swabbing impossible. Samples were stored in a 0.9% sodium saline solution containing 10 mg/L chloramphenicol at 4 °C. Once 10 to 20 samples (15 on average) were gathered over one week, they were sent the next Monday by postal mail to the Laboratoire de Parasitologie et Mycologie Médicale for processing. The transport delay ranged from 2 to 4 days, with a mean time of 3 days.

### 2.4. Treatment of Samples

Upon reception, samples were treated as per the following protocol adapted from the standardized protocol used by the International Society for Human and Animal Mycology (ISHAM) Cryptococcal Working Group [7]; in brief, they were seeded on 2 Niger Seed agar plates (supplemented with 20 U/mL penicillin, 40 U/mL gentamycin, 0.25 mg/mL chloramphenicol, and 5 mg/mL benomyl) and left to grow separately at 25 °C and 35 °C for 7 days. Each possible *Cryptococcus* colony, selected by examination based on the phenotypic characteristics and color of the colony, was checked for the presence of a capsule by India ink staining and tested for its urease capability (Biomerieux, Marcy l’Etoile, France). Colonies with a capsule and positivity on the urease test were characterized biochemically with an API ID 32C strip.

### 2.5. Genomic Amplification

#### 2.5.1. DNA Extraction

Genomic DNA was extracted for each strain using a NucleoSpin blood quick extraction kit (Macherey-Nagel Gmb and Co. KG, Duren, Germany) with modifications as previously described [47].

#### 2.5.2. Amplification

Genomic amplification was performed with the universal panfungal primers ITS1 (5′-TCCGTAGGTGAACCTGCGG-3′) and ITS4 (5′-TCCTCCGCTTATTGATATGC-3′) [48]. The reaction mix was as follows: MgCl2 1.5 mM, dNTPs 200 µM, primer ITS1 0.2 µM, primer ITS4 0.2 µM, Taq polymerase 0.5 U, 100 ng of DNA and 1x reaction buffer. Runs were performed with the following cycle: 95 °C for 5 min; 35 cycles of 95 °C, 1 min; 60 °C for 1 min; and 72 °C for 1 min; followed by a final step at 72 °C for 5 min.

#### 2.5.3. Sequence Analyses

Sequencing of the amplicons was performed by Genewiz (Azenta Life Sciences, Leipzig, Germany) on both strands (forward and reverse) using IT1 and ITS4 primers for sequencing. Sequences were compared to the global NCBI standard nucleotide collection database (nr/nt) by BLAST. Identification was considered acceptable when a hit was obtained with an *E*-value of 0.0, a query coverage of at least 99%, homology of 100% for one strand, and at least ≥99.45% for the other strand (only if the mismatches occurred in or next to the sequence priming or termination areas). When species discrimination could not be achieved with ITS1-ITS4 fragment sequencing, further amplification with the 28 S large nuclear subunit primers NL1 (5′GCATATCAATAAGCGGAGGAAAAG-3′) and NL4 (5′-GGTCCGTGTTTCAAGACGG-3′) [49] was performed and processed as described above.

### 2.6. Statistical Analyses

Fisher’s exact test for count data with simulated p-values (based on 2000 replicates) was used to assess whether there were associations of bird species, order, age, clinical condition, and treatment with positivity for RCFC.

The chi^2^ test was used to assess whether there was an association between the type of sampling and positivity for RCFC.

The chi^2^ test was used to determine whether there was an association between antibiotic treatment, use of a nonsteroidal anti-inflammatory drug (NSAID, meloxicam) or steroidal anti-inflammatory drug (rapidexon), or any combination of these drugs and positivity for RCFC.

The chi^2^ test was used to determine if birds’ diet had an impact on positivity for RCFC and RCFC species distribution. For the analysis, birds were classified into seven groups according to their main diet at the adult stage: grain eaters, fruit eaters, small vertebrate eaters, insectivores, omnivorous birds, opportunistic feeders, and, finally, others. Grain eaters group corresponds to birds feeding mainly on grain and/or seeds; fruit eaters group corresponds to birds feeding mainly on fruit and/or berries. The small vertebrate eaters group includes birds feeding on a variety of small terrestrial vertebrates, such as mice, voles, small reptiles, and small passerine birds. Insectivore group contains birds feeding on a wide variety of insects and other terrestrial arthropods such as spiders. The omnivorous feeders group contains birds with no preferential diet between plants/grains and animals. Opportunistic feeders group corresponds to birds able to feed on a wide variety of food sources including human wastes. Finally, the other group includes all birds whose diet does not fit into any of the previous groups; specialist feeders, birds feeding on invertebrates other than insects such as annelids, molluscs, or crustaceans, birds feeding on aquatic food sources such as fishes or amphibians, carrion eaters, etc.

Birds with two different eating habits (for example, grain eater but also fruit eater) were counted once in each group for the statistical analysis.

## 3. Results

### 3.1. Cryptococcal Species Distribution in the Bird Population

#### 3.1.1. Total Cryptococcal Species Distribution

One hundred and seven of the 669 birds (16%) belonging to 15 different orders and 37 different species were positive for at least one RCFC species. No statistical correlation was found between bird orders or species and positivity for yeasts (*p* = 0.64 and *p* = 0.9, respectively) (Table 1 and Supplementary Data S2).

#### 3.1.2. Assessment of Highly Pathogenic *Cryptococcus* Species

No species belonging to the highly pathogenic *C. neoformans* or *C. gattii* species complexes were found in the bird population sampled.

#### 3.1.3. Rare *Cryptococcus* and Former *Cryptococcus* Species Diversity

Regarding the distribution of RCFC species among the positive birds, ninety-eight (91.5%) birds were positive for a single species, while nine (8.5%) harbored two different species. Thus, the total number of RCFCs found in our study was 116. Molecular analyses identified 20 different species in the bird population. One isolate could not be identified with certainty at the species level and is considered undetermined. The species identified were as follows (Table 2):

Cutaneotrichosporon curvatus (3), Cryptococcus ovalis (2), Filobasidium chernovii (2), Filobasidium floriforme (4), Filobasidium magnum (36), Filobasidium oierense (2), Filobasidium wieringae (1), Holtermanniella festucosa (1), Naganishia adeliensis (1), Naganishia albida (16), Naganishia diffluens (12), Naganishia globosa (5), Naganishia liquefasciens (2), Naganishia randhawae (1), Naganishia uzbekistanensis (8), Papiliotrema flavescens (3), Papiliotrema laurentii (3), Papiliotrema terrestris (2), Solicoccozyma aeria (2), Vishniacozyma carnescens (9) and Cryptococcus sp. (1).

The distribution of positive bird species can be found in Table 1 and Supplementary Data S2.

**Figure 1:**
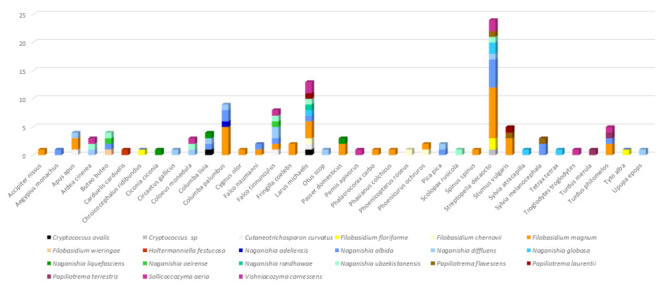
Distribution of *Cryptococcus* and former *Cryptococcus* species by bird of origin.

The distribution of yeast species according to their bird of origin can be found in Figure 1 and Table 2.

### 3.2. Distribution of RCFC in Birds in Regard of Method of Sampling, Age, Illnesses, Medications and Diets

#### 3.2.1. Distribution by Type of Sampling

**Figure 2:**
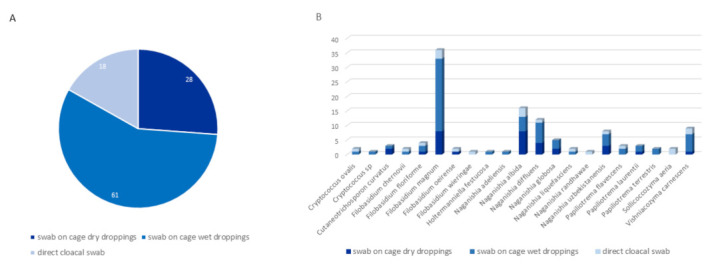
Distribution of samples positive for rare *Cryptococcus* and former *Cryptococcus* spp. (**A**): Overall distribution of positive samples by type of sampling. (**B**): Distribution of *Cryptococcus* and former *Cryptococcus* species by type of sampling.

Most samples positive for RCFC were retrieved from cage swabs of wet feces (61/107; 57%), followed by cage swabs of dry feces (28/107; 26.2%) and finally cloacal swabs (18/107; 16.8%), as shown in Figure 2. According to the chi^2^ test, there was a significant (*p* = 0.0002) association between the sampling site and the positivity of samples. Indeed, there is an overrepresentation of positive dry dropping swabs in comparison to wet dropping swabs and direct cloacal swabs (Figure 2 and Supplementary Data S3).

#### 3.2.2. Distribution by Age

Ninety-four of the birds positive for RCFC were adults (total adults 486, 19.3%), eight were juveniles (total juveniles 132, 6%) and five were immatures (total immatures 41, 12.2%). A significant (*p* = 0.0005, Fisher’s exact test) association was observed between age class and RCFC positivity in birds. The most significant difference was between adults and juveniles. Indeed, under the null hypothesis of independence, the expected number of positive samples was 77.7 and 21.1 for these respective age classes, whereas the observed number of positive samples was 94 and 8. Therefore, an over-representation of adults and an under-representation of juveniles were observed among the positive birds (Table 3 and Supplementary Data S3).

#### 3.2.3. Distribution by Health Issue

Eleven of 107 birds (11.3%) had a reported health issue, including the presence of lead shot (5/11), an infectious disease (5/11), or electrocution (1/11). The other 96 birds had no reported diseases or wounds. According to Fisher’s exact test, there was no significant (*p* = 0.48) association between any documented health condition and positivity for RCFC. The power of the test was weak, so rare conditions (<5%) were grouped together, but Fisher’s exact test remained nonsignificant (*p* = 0.52). Health conditions were grouped further (physical/trauma ailments or infectious diseases), but there was still no significant correlation with bird positivity (*p* = 1) (Table 3 and Supplementary Data S3).

Notably, no bird had documented symptoms of fungal infection, including symptoms of cryptococcal infection (Table 3).

#### 3.2.4. Distribution by Medical Treatment

Fourteen birds positive to RCFCs, including twelve for which no wounds or diseases were reported, had a history of drug administration, mostly a combination of antibiotics (amoxicillin or amoxicillin + clavulanate) and anti-inflammatory drugs (meloxicam or rapidexon). According to Fisher’s exact test, there was a nonsignificant (*p* = 0.89) association between any treatment and positivity for RCFC. However, the power of the test was weak. To increase the power of the test, some rare treatments (<5%) were grouped. However, Fisher’s exact test remained nonsignificant (*p* = 0.36). We thus focused on the treatments that increased susceptibility to yeast carriage (antibiotics, NSAIDs, steroidal anti-inflammatory drugs, and any combination of these three drugs), but again, no statistically significant correlation was found (*p* = 0.61, *p* = 0.33, *p* = 1 and *p* = 1, respectively) (Table 3 and Supplementary Data S3).

#### 3.2.5. Distribution of RCFC Species according to Their Carrier Bird Feeding Habits

The distribution of birds positive to RCFC according to their diet was as follows:

Thirty-seven grain eaters (total grain eaters 208, 17.8%); 17 fruit (total berries eaters 71, 23.9%), 15 small vertebrate eaters (total small vertebrate eaters 109, 13.7%), 17 insectivorous birds (total insectivores 88, 19.3%), 27 omnivorous birds (total omnivorous birds 201, 13.4%), 23 opportunistic feeders (total opportunistic feeders 169, 13.6%) (Table 4).

Chi^2^ analysis of positivity for RCFC with regard to feeding habits showed that there was no correlation between the two elements (*p* = 0.26).

No correlation was found between the feeding habit and the RCFC species distribution among the birds (*p* = 0.78).

## 4. Discussion

### 4.1. RCFC Diversity

Studies of the carriage of *Cryptococcus* and species formerly belonging to the *Cryptococcus* genus in free-ranging wild birds are rare because they are technically challenging as capture needs to be done without harming the animal. Additionally, a significant number of wild birds are considered protected species in Europe. To circumvent these challenges and to assess whether wild birds can act as carriers or spreaders of the most pathogenic *Cryptococcus* species (*Cryptococcus neoformans/gattii s.l.*) in southern France, we sampled animals admitted for care at a care center managed by the French Bird Protection League. No yeasts belonging to the highly pathogenic *C. neoformans/C. gattii s.l.* complexes were found during this study. This seems contradictory to various studies suggesting that southern France, where we performed the sampling, should harbor these two species [50,51] but is in accordance with literature reporting that isolation of *C. neoformans* in wild birds is rare [52]. On the other hand, the RCFC diversity in the bird population we sampled was high, with twenty different species isolated. Among them, one species (*S. aeria*) has never been isolated from any animal source. Five others, namely, *Cryptococcus ovalis*, *N. chernovii*, *H. festucosa*, *F. oeirense*, and *F. wieringae*, were previously isolated from various infected or asymptomatic mammals, such as cats, dogs, or horses, but have not been isolated from any birds [40,53,54]. The fourteen remaining species had already been isolated from bird sources but mostly from pigeon droppings [55,56,57,58] and captive or pet birds [59,60,61,62]. In fact, very few wild birds have been described as possible sources for RCFC. *N. adeliensis* and *F. magnum* were previously isolated from a cormorant [63]. *N. albida* was isolated from a number of wild ducks and teals [63]. *P. laurentii* was also isolated from a very limited number of migratory birds, Canadian goose feces, a bird of prey, and a house sparrow [64,65,66]. *N. globosa* was isolated from a bird belonging to the Charadriiforme order [67]. By contrast, we found that RCFC species were distributed in fifteen bird orders, thirty-four bird genera, and thirty-seven bird species. Thus, most of the RCFC species identified in this study have not been previously associated with the source birds described herein.

This lack of information about RCFC carriage in birds could be explained in two ways. First, a significant number of RCFC species are considered to be rare. Furthermore, most studies on *Cryptococcus* focus on the most medically important species; *C. neoformans* and *C. gattii s.l.*, and thus limit themselves to the known reservoirs of these species i.e., pigeon droppings in urban areas and tree hollows [46]. Very few studies expand sampling to a wider variety of birds, and when doing so, it is mainly limited to captive or pet birds [59]. However, urban environments and captivity constitute specific situations that can influence the microbial composition of the animals tested and thus lead to possible bias. Indeed, population density, environmental stress, and food access in cities or in captivity are very different from those in the wild. It has been shown that proximity to humans and human activities influence the number and variety of yeasts present in bird feces [68]. It has also been described that nutritional status influences bird susceptibility to various pathogens [69]. Finally, it has been shown that bird density, as well as the cage and environmental hygiene, play significant roles in the presence of microbial pathogens in bird feces and/or in the shedding of pathogens by animals [70,71]. Therefore, focusing on pigeons in urban areas (or on captive or pet birds) may lead to a lack of data on the non-*neoformans*/non *gattii* RCFC possible reservoirs. We thus suggest that any study on RCFC yeasts should, when possible, expand sampling beyond usual urban Columbiformes (pigeon droppings and nests) sources.

### 4.2. Distribution of RCFC According to Bird Orders, Species, Diet, Clinical Data, Age and Type of Sampling

No significant correlations between order, species, or feeding habit/diet and RCFC carriage were found in this study. RCFC species distribution shows that the same yeast species can be shared by different carrier birds with different ecological niches and feeding habits. For example, *N. albida* was present in birds of prey, carrion eaters, opportunistic feeders, grain eaters, and insectivorous birds. *N. diffluens* was present in small vertebrate eaters, fish eaters, insectivores, and omnivorous and opportunistic birds, such as magpies and jackdaws. *F. magnum* was found in birds of prey, grain eaters, berry eaters and fish eaters. This result suggests that RCFC species may be present through the whole trophic chain from plants to invertebrates [72] to free-ranging small vertebrates [73] that serve as bird food. Alternatively, it is possible that bird carriage of RCFC species is not linked to their feeding regimen but to contact with an external source of contamination, such as decomposing plant material, where some RCFC can be found [74].

There was no significant correlation between recorded health issues or medical treatment and RCFC carriage. In particular, the intake of drugs, including antibiotics, NSAIDs and steroid anti-inflammatory drugs, which are known to facilitate fungal colonization, had no influence on sample positivity in our study.

On the other hand, a correlation between yeast carriage and bird age was found. Adult birds were over-represented in the RCFC-positive animals, while juveniles were under-represented. Studies on the association between *Cryptococcus* sp. (or species formerly belonging to the *Cryptococcus* genus) carriage and animal age are rare. When performed in cats and koalas, age was not a determinant of carriage [40,75]. It has been, however, shown that colonization with other fungi (mainly *Candida krusei* and *Candida albicans*) increases with age in house sparrows [66], suggesting a cumulative risk of carriage with age. Our data seem to be in accordance with the latter result. However, this needs confirmation because our cohort of aged birds was small, with only ten individuals labeled as such and none of them positive for RCFC.

A correlation between the method of sampling and positivity to RCFC was also observed in this study. Indeed, dry cage swabs were over-represented in comparison with wet cage and direct cloacal swabs among positive birds. This is comparable with what is known about the growth of the most studied *Cryptococcus* species; *Cryptococcus neoformans s.l.*: (i) the growth of these yeasts are inhibited in a medium undergoing alkalinization due to bacterial growth, such as wet droppings [76]; and (ii) dry droppings have a higher concentration of key nutrients, such as urea and creatinine, and are thus supposedly a more favorable growth medium for the yeasts [77]. On the other hand, RCFC isolates were also found in wet droppings, while C. *neoformans/C. gattii s.l.* species have almost never been isolated from such media [78]. The fact that some RCFC species, particularly *Naganishia* spp., seem to be able to survive in environments poor in carbon and nitrogen and with an alkaline or acidic pH, situations that would be hostile to *C.* *neoformans/C. gattii s.l.*, may explain this last observation [79].

### 4.3. The Particular Case of Direct Cloacal Swabs

In our study, fourteen different RCFC species were isolated from eighteen birds sampled by direct cloacal swabs. This excludes the possibility of ground or cage contamination of the samples and suggests that the yeasts may come from the digestive tract of the animals. Notably, two species, *C. ovalis,* and *S. aeria*, were found by such method of sampling, suggesting that they can pass through birds’ digestive system, while they were never isolated from any bird source before. However, none of *C. ovalis* or *S. aeria* isolates found in this study grew at a temperature of 35 °C after seeding on Niger seed agar plates, but only at 25 °C. One possible explanation is that the yeasts originated in the environment and were accidentally present on the feathers around the cloaca of the birds. According to this hypothesis, detection by cloacal swabbing may be due to contamination of the swabs by contact with colonized feathers. To test this hypothesis, attempts to grow these two species at 30 °C, a temperature that is below the bird’s skin temperature, were made. *S. aeria*, but not *C. ovalis*, showed (poor) growth at 30 °C, suggesting a possible bird feathers origin for this species. On the other hand, no *S. aeria* was found by sampling methods inducing a risk of contamination from the environment (cage swabs). The two different samples positive for *S. aeria* obtained during this study were found only by cloacal swabs, raising questions about the true colonization site in birds: feathers or digestive tract, for this species. The twelve other species isolated from bird cloaca (*F. chernovii*, *F. floriforme*, *F. magnum*, *F. wieringae*, *N. adeliensis*, *N. albida*, *N. liquefasciens*, *N. randhawae*, *N. uzbekistanensis*, *P. flavescens*, *V. carnescens*) have all been previously isolated from pigeon sources or from other pet/captive birds but only from cages, nests, or dry ground droppings. The fact that these species were found in cloacal samples suggests an ability to survive in the birds’ digestive tract too. However, as previously observed for *C. ovalis*, none of the isolates showed thermotolerance in culture. Thus far, we have no explanation for the discrepancy between species retrieved from a source with a high-temperature body and the lack of thermotolerance exhibited in culture unless the aforementioned species share the same escape mechanisms described for *C. neoformans.* Indeed, it was shown that *C. neoformans* can pass through the complete intestinal system of birds [80,81,82]. While this passage requires adaptation to the high body temperature of birds, it is possible for a minority of *C. neoformans* cells by vomocytosis or cell enlargement [83]. However, to our knowledge, no such mechanisms were yet described for any RCFC. Nevertheless, the proportion of animals positive for RCFC by cloacal swab appears high, particularly in comparison with what can be achieved for *C. neoformans/C. gattii s.l.* For these species’ complexes, positivity by cloacal swab is rare in literature and ranges from 0% to 4.8% at most [52], while we observed 16.8% RCFC positivity in the bird population we studied. Furthermore, some cloacal swab samples were positive for two different RCFC, suggesting that different species can survive simultaneously in the same bird digestive system. Infection by up to three different *Cryptococcus neoformans/gattii s.l.* species in the same patient has already been described in humans [47]; but the carriage of multiple RCFC species in the digestive tract of the same carrier (or reservoir) animal has never been described so far.

### 4.4. Risk Assessment for Humans

Concerning the risk assessment for humans, including caregivers in protection and sheltering centers, no highly pathogenic species, such as *C. neoformans s.l.*, were found in this study, even in Columbiformes (pigeons and doves) that are known reservoirs for this pathogen. Isolation of *C. neoformans* has been documented in the literature in a limited number of other birds than pigeons, such as *Anas crecca*, *A. platyrhinchos* [52,63], *Falco tinnunculus* [64], and various passerine birds [66,84]. However, the positive samples obtained in these studies came in fact from dead birds, birds in an aviary, or birds captured on buildings in an urban area or in villages. Although the same bird species are present in our bird cohort, they were negative for *C. neoformans* in our study. Very few other studies were performed in exclusively wild populations, and they did not detect *C. neoformans* either [85]. These observations may cast doubt on the presence of *C. neoformans s.l.* in wild fauna, where stress, population concentration, range, and access to food are different from those in captured birds or flocks of birds living in urbanized or anthropogenized areas. Nevertheless, this study shows that the risk of exposure to highly pathogenic *Cryptococcus* species in nonurban human populations and bird caretakers is limited. For the less pathogenic RCFC species, particularly those already known to infect humans [5], the risk may be elevated in care personnel working in aviaries or wild fauna protection centers. The risk may also be present for peri/suburban human populations because residential gardens are attractive to some carrier birds, particularly Passeriformes. However, given that RCFC rarely causes clinically significant infections in humans [86], the overall probability of infection seems very low but not impossible.

Conclusion: In this study, owing to the diversity of our study population, we elucidated the relationship between various bird species and the carriage of RCFC species. A large number of the reported relationships of RCFC species and the birds they were isolated from have not been previously described in the literature. Thus, this study may help to clarify the role of wild birds as ecological niches for rare *Cryptococcus* and species formerly belonging to the *Cryptococcus* genus.

## Figures and Tables

**Table 1:** Distribution of bird Orders and species positive to rare or former *Cryptococcus* (RCFC) yeasts.

Bird Order	Total Birds Sampled within the Order	Number of Birds Positive to RCFC within the Order	Individual Species Names for Positive Birds	Common Names for Positive Birds	Total Number of Individuals Sampled within the Species	Number of Positive Individuals within the Species
Accipitriformes	75	8 (10.6%)	*Buteo buteo*	Common buzzard	43	4 (9.3%)
			*Accipiter nisus*	European sparrowhawk	20	1 (5%)
			*Aegypius monachus*	Cinereous vulture	1	1 (100%)
			*Circaetus gallicus*	Short toed snake eagle	2	1 (50%)
			*Pernis apivorus*	European honey buzzard	1	1 (100%)
Anseriformes	17	1 (5.9%)	*Cygnus olor*	Mute swan	2	1 (50%)
Apodiformes	14	3 (21.4%)	*Apus apus*	Common swift	12	3 (25%)
Bucerotiformes	8	1 (12.5%)	*Upupa epops*	Eurasian hoopoe	8	1 (12.5%)
Charadriiformes	113	15 (13.3%)	*Larus michaelis*	Yellow-legged gull	100	13 (13%)
			*Chroicocephalus ridibundus*	Black Headed gull	10	1 (10%)
			*Scolopex rusticola*	Eurasian woodcock	2	1 (50%)
Ciconiformes	5	1 (20%)	*Ciconia ciconia*	White stork	3	1 (33.3%)
Columbiformes	180	33 (18.3%)	*Streptopelia decaocto*	Eurasian collared dove	127	20 (15.7%)
			*Columba palumbus*	Common wood pigeon	27	9 (33.3%)
			*Columba livia*	Common pigeon/rock pigeon	23	4 (17.4%)
Falconiformes	32	9 (28.1%)	*Falco tinnunculus*	Common kestrel	25	7 (28%)
			*Falco naumanni*	Lesser kestrel	4	2 (50%)
Galliformes	8	1 (12.5%)	*Phasianus colchicus*	Common pheasant	1	1 (100%)
Otidiformes	1	1 (100%)	*Tetrax tetrax*	Little bustard	1	1 (100%)
Passeriformes	153	27 (17.6%)	*Sturnus vulgaris*	Common starling	22	5 (22.7%)
			*Turdus philomelos*	Song thrush	10	4 (40%)
			*Passer domesticus*	House sparrow	19	3 (15.8%)
			*Coeloeus monedula*	Eurasian jackdaw	16	2 (12.5%)
			*Fringilla coelobs*	Common chaffinch	6	2 (33.3%)
			*Phoenicurus ochruros*	Black redstart	4	2 (50%)
			*Pica pica*	Eurasian magpie	24	2 (8.3%)
			*Sylvia melanocephala*	Sardinian warbler	3	2 (66.6%)
			*Carduelis carduelis*	European goldfinch	4	1 (25%)
			*Spinus spinus*	Eurasian siskin	2	1 (50%)
			*Sylvia atricapilla*	Eurasian blackcap	5	1 (20%)
			*Troglodytes troglodytes*	Eurasian wren	1	1 (100%)
			*Turdus merula*	Common blackbird	2	1 (50%)
Pelecaniformes	7	3 (42.8%)	*Ardea cinerea*	Grey heron	5	3 (60%)
Phoenicopteriformes	6	1 (16.6%)	*Phoenicopterus roseus*	Greater flamingo	6	1 (16.6%)
Strigiformes	33	2 (6%)	*Otus scops*	European scops owl	6	1 (16.6%)
			*Tyto alba*	Barn owl	3	1 (33.3%)
Suliformes	6	1 (16.6%)	*Phalacrocorax carbo*	Great cormoran	3	1 (33.3%)

**Table 2:** Distribution of rare *Cryptococcus* and former *Cryptococcus* (RCFC) species by numbers found during the study and corresponding birds (Orders and species) they were isolated from. Numbers in brackets indicate the number of individual birds within an order or a specie the corresponding RCFC were isolated from.

RCFC Species	Numbers Isolated	Birds from Which RCFC Were Isolated (Order)	Birds from Which RCFC Were Isolated (Species)
*Cryptococcus ovalis*	2	Columbiforme (1) Chaaradriforme (1)	*Columba livia* (1) *Larus michaelis* (1)
*Cryptococcus* sp.	1	Columbiforme (1)	*Streptopelia decaocto* (1)
*Cutaneotrichosporon curvatus*	3	Falconiforme (1) Apodiforme (1) Charadriiforme (1)	*Falco tinnunculus* (1) *Apus apus* (1) *Larus michaelis* (1)
*Filobasidium chernovii*	2	Phoenicoptiforme (1) Passeriforme (1)	*Phoenicopterus roseus* (1) *Phoenicurus ochruros* (1)
*Filobasidium floriforme*	4	Columbiforme (2) Charadriiforme (2) Strigiforme (1)	*Chroicocephalus ridibundus* (1) *Streptopelia decaocto* (2) *Tyto alba* (1)
*Filobasidium magnum*	36	Accipitriforme (1) Apodiforme (2) Columbiforme (15) Falconiforme (2) Passeriforme (11) Charadriiforme (3) Suliforme (1) Galliforme (1)	*Accipiter nisus* (1) *Apus apus* (2) *Columba livia* (1) *Columba palumbus* (5) *Falco naumanni* (1) *Falco tinnunculus* (1) *Fringilla coelebs* (2) *Larus michaelis* (3) *Passer domesticus* (2) *Phalocrocorax carbo* (1) *Phasianus colchicus* (1) *Phoenicurus ochruros* (1) *Spinus spinus* (1) *Streptopelia decaocto* (9) *Sturnus vulgaris* (3) *Turdus philomelos* (2)
*Filobasidium wieringae*	1	Accipitriforme (1)	*Buteo buteo* (1)
*Holtermanniella festucosa*	1	Passeriforme (1)	*Carduelis carduelis* (1)
*Naganishia adeliensis*	1	Columbiforme (1)	*Columba palumbus* (1)
*Naganishia albida*	20	Accipitriforme (2) Columbiforme (6) Charadriiforme (1) Falconiforme (3) Passeriforme (4)	*Aegypius monachus* (1) *Columba livia* (1) *Streptopelia decaocto* (5) *Buteo buteo* (1) *Falco naumanni* (1) *Falco tinnunculus* (2) *Larus michaelis* (1) *Pica pica* (1) *Sylvia melanocephala* (2) *Turdus philomelos* (1)
*Naganishia diffluens*	12	Apodiforme (1) Pelecaniforme (1) Accipitriforme (1) Passeriforme (2) Falconiforme (2) Charadriiforme (1) Strigiforme (1) Columbiform (2) Bucerotiforme (1)	*Apus apus* (1) *Ardea cinerea* (1) *Circaetus gallicus* (1) *Coloeus monedula* (1) *Columba palumbus* (1) *Falco tinnunculus* (2) *Larus michaelis* (1) *Otus scops* (1) *Pica pica* (1) *Streptopelia decaocto* (1) *Upupa epops* (1)
*Naganishia globosa*	5	Charadriiforme (1) Columbiforme (2) Passeriforme (1) Otidiforme (1)	*Larus michaelis* (1) *Streptopelia decaocto* (2) *Sylvia atracapilla* (1) *Tetrax tetrax* (1)
*Naganishia liquefasciens*	2	Ciconiiforme (1) Passeriforme (1)	*Ciconia ciconia* (1) *Passer domesticus* (1)
*Naganishia oeirense*	2	Accipitriforme (1) Falconiforme (1)	*Buteo buteo* (1) *Falco tinnunculus* (1)
*Naganishia randhawae*	1	Charadriiforme (1)	*Larus michaelis* (1)
*Naganishia uzbekistanensis*	8	Pelecaniforme (1) Accipitriforme (1) Columbiforme (2) Falconiforme (1) Charadriiforme (2) Passeriforme (1)	*Ardea cinerea* (1) *Buteo buteo* (1) *Columba livia* (1) *Falco tinnunculus* (1) *Larus michaelis* (1) *Scolopax rusicola* (1) *Streptopelia decaocto* (1) *Coelus monedula* (1)
*Papiliotrema flavescens*	3	Passeriforme (2) Columbiforme (1)	*Sylvia melanocephala* (1) *Sturnus vulgaris* (1) *Streptopelia decaocto* (1)
*Papiliotrema laurentii*	3	Columbiforme (1) Charadriiforme (1) Passeriforme (1)	*Columba palumbus* (1) *Larus michaelis* (1) *Sturnus vulgaris* (1)
*Papiliotrema terrestris*	2	Passeriformes (2)	*Turdus philomelos* (1) *Turdus merula* (1)
*Solicoccozyma aeria*	2	Columbiforme (1) Passeriforme (1)	*Streptopelia decaocto* (1) *Turdus philomelos* (1)
*Vishniacozyma carnescens*	9	Passeriforme (2) Columbiforme (2) Pelecaniforme (1) Charadriiforme (2) Anseniforme (1) Accipitriforme (1)	*Coelus monedula* (1) *Ardea cinerea* (1) *Columba palumbus* (1) *Cygnus olor* (1) *Larus michaelis* (2) *Pernus apivorus* (1) *Troglodytes troglodytes* (1) *Streptopelia decaocto* (1)

**Table 3:** General data (age distribution, documented clinical and medication data) about the sampled bird population.

	Total Birds (669)	Positive Birds to RFCF (107, 16%)
**Age**		
Aged birds Adults Juveniles Immatures	10 486 132 41	0 94 (19.3%) 8 (6%) 5 (12.2%)
**Birds with documented clinical data**	69	11 (16%)
*Infectious diseases* Trichomonosis Botulism Bot fly Coccidiosis Avian pox Newcastle disease Ocular infection Digestive infection/parasites	29 6 8 1 3 6 2 1 2	5 (17.2%) 0 1 (12.5%) 0 1 (33.3%) 1 (16.6%) 2 (100%) 0 0
*Trauma or physiological trouble* Wounds by lead pellets Electrocution Neurological shock Internal bleeding Hematoma Poisoning Trauma Oiled birds Cachexia	40 14 7 7 3 2 1 1 2 3	6 (15%) 5 (35.7%) 1 (14.3%) 0 0 0 0 0 0 0
**Birds with documented treatments**	90	14 (15.5%)
Amoxicillin + clavulanate Amoxicillin + clavulanate +Rapidexon Amoxicillin + clavulanate + Meloxicam Rapidexon Meloxicam Thiamine-Pyridoxine + Vincamine-papaverine Thiamine-Pyridoxine + Vincamine-papaverine + Rapidexon Thiamine-Pyridoxine + Vincamine-papaverine + Meloxicam + vitamin K Rapidexon + Etamsylate Ocular oilment with chloramphenicol Activated coal Fortol (enriched recovery liquid) Activated coal + Etamsylate Rapidexon + Meloxicam + Etamsylate Ocular oilment with oxytetracyline and dexamethasone Carnidazole + Meloxicam Etamsylate + Meloxicam Glucose Rapidexon + Meloxicam Thiamine-Pyridoxine + Vincamine-papaverine + Meloxicam + Rapidexon Etamsylate	8 3 19 11 17 3 2 1 3 2 4 2 4 1 1 1 2 2 2 1 1	1 (12.5%) 0 5 (26.3%) 1 (9%) 4 (23.5%) 0 1 (50%) 0 0 0 1 (25%) 0 0 1 (100%) 0 0 0 0 0 0 0

**Table 4:** Distribution of birds according their main diet at adult stage. Grain eaters indicates birds feeding mainly on grain and/or seeds at adult stage; Fruit eaters indicates birds feeding mainly on fruit and/or berries at adult stage; Small vertebrate eaters indicates birds feeding on a variety of small terrestrial vertebrate preys such as mice, voles, small reptiles and small birds at adult stage; Insectivore indicates birds feeding on a wide variety of insects and other terrestrial arthropods such as spiders at adult stage; Omnivorous feeders indicates birds with no preferential diet between plants/grain and animals; Opportunistic feeders indicates birds with a wide variety of food sources that also scavenge on human wastes; Other group indicates birds with a diet that does not fit into any of the previous groups. This includes specialist feeders, birds feeding on invertebrates other than insects such as annelids, molluscs or crustaceans, birds feeding on aquatic food sources such as fishes or amphibians. Birds with several main diets (for example grain eater and fruit eaters) were counted once in each category.

Main Diet Category at Adult Stage	Total Number of Birds in the Diet Category	Birds Positive to RCFC in the Diet Category
Grain eaters	208	37 (17.8%)
Fruits eaters	71	17 (23.9%)
Small vertebrates eaters	109	15 (13.7%)
Insectivores	88	17 (19.3%)
Omnivorous	201	27 (13.4%)
Opportunistic feeders	169	23 (13.6%)
Others	69	11 (15.9%)

## Data Availability

Data are contained within the article or supplementary material. No sequences or strains were submitted to any database or library. However, they are accessible upon request.

## Supplementary Materials

The following supporting information can be downloaded at: https://www.mdpi.com/article/10.3390/jof8030227/s1, Supplementary Data S1: Map indicating the position of Montpellier city, the position of the Centre de Sauvegarde de la Faune Sauvage. Areas labelled in red show the areas where the birds admitted to the Centre de Sauvegarde de la Faune Sauvage were retrieved from. Supplementary Data S2: Complete list of birds Orders and species sampled during the study and number of individuals positive to rare *Cryptococcus* and former *Cryptococcus* (RCFC) within each species. Supplementary Data S3: Detailed Distribution of rare *Cryptococcus* and former *Cryptococcus* species by bird of origin and method of sampling. A: indicates an Adult; J: indicates a Juvenile; I: indicates an Immature bird. ND indicates no specific clinical data. ITS indicates that sequences were obtained by ITS1-ITS4 sequencing. NL indicates that sequences were obtained by NL1-NL4 sequencing.
