# Supplementary material for: Search for Cryptococcus neoformans/gattii Complexes and Related Genera (Filobasidium, Holtermanniella, Naganishia, Papiliotrema, Solicoccozyma, Vishniacozyma) spp. Biotope: Two Years Surveillance of Wild Avian Fauna in Southern France
Source: J Fungi (Basel). 2022 Feb 24;8(3):227. doi: 10.3390/jof8030227 (PMC8948691; doi:10.3390/jof8030227)
Supplement: Supplementary file 1 [file jof-08-00227-s001.zip › Supplementary Data S2.pdf]

Order	Individual birds names	Individual birds common names	Main feeding diet at adult stage	Total number of individuals sampled within the specie	Number of positive individuals within the specie
Accipitriformes	*Buteo buteo*	Common buzzard	Small vertebrates eater	43	**4 (9.3%)**
	Accipiter nisus	European sparrowhawk	Other	20	**1 (5%)**
	Aegypius monachus	Cinereous vulture	Other	1	**1 (100%)**
	Circaetus gallicus	Short toed snake eagle	Other	2	**1 (50%)**
	Pernis apivorus	European honey buzzard	Other	1	**1 (100%)**
	Accipiter gentilis	Goshawk	Small vertebrates eater	1	0
	Aquila chrysaetos	Golden eagle	Other	1	0
	Circus aeruginosus	Western marsh harrier	Small vertebrates eater	1	0
	Gypaetus barburus	Bearded vulture	Other	1	0
	Gyps fulvus	Griffon vulture	Other	2	0
	Hieraaetus pennatus	Booted eagle	Small vertebrates eater	1	0
	Milvus migrans	Black kite	Small vertebrates eater	1	0
Anseriformes	*Cygnus olor*	Mute swan	Other	2	**1 (50%)**
	Anas creca	Common teal	Other	1	0
	Anas platyrhynchos	Mallard duck	Omnivorous	12	0

	Tadorna tadorna	Common shelduck	Other	2	0
Apodiformes	*Apus apus*	Common swift	Insectivorous	12	**3 (25%)**
	Apus pallidus	Pallid swift	Insectivorous	2	0
Bucerotiformes	*Upupa epops*	Eurasian hoopoe	Insectivorous	8	**1 (12.5%)**
Caprimulgiformes	*Caprimulgus europaeus*	Eurasian nightjar	Insectivorous	1	0
Charadriiformes	*Larus michaelis*	Yellow-legged gull	Omnivorous, Opportunistic	100	**13 (13%)**
	Chroicocephalus ridibundus	Black Headed gull	Omnivorous, Opportunistic	10	**1 (10%)**
	Scolopex rusticola	Eurasian woodcock	Other	2	**1 (50%)**
	Burhinus oedicnemus	Eurasian thick-knee/ stone-curlew	Other	1	0
Ciconiiformes	*Ciconia ciconia*	White stork	Other	3	**1 (33.3%)**
	Egretta garzetta	Little egret	Other	2	0
Columbiformes	*Streptopelia decaocto*	Eurasian collared dove	Grain eater	127	**20 (15.7%)**
	Columba palumbus	Common wood pigeon	Grain eater, fruit eater	27	**9 (33.3%)**
	Columba livia	Common pigeon/ rock pigeon	Grain eater, fruit eater, Opportunistic feeder	23	**4 (17.4%)**
	Columba livia domestica	Common pigeon/ rock pigeon	Grain eater, fruit eater, Opportunistic feeder	1	0

	Columba sp	XXX	Grain eater, fruit eater	2	0
Coracciformes	*Alcedo atthis*	Common kingfisher	Other	3	0
	Merops apiaster	European bee eater	Insectivorous	1	0
Cuculiformes	*Clamator glandarius*	Great spotted cuckoo	Insectivorous	4	0
Falconiformes	*Falco tinnunculus*	Common kestrel	Small vertebrates eater	25	**7 (28%)**
	Falco naumanni	Lesser kestrel	Small vertebrates eater, Insectivorous	4	**2 (50%)**
	Falco peregrinus	Peregrine falcon	Other	1	0
	Falco subbuteo	Eurasian hobby	Other	2	0
Galliformes	*Phasianus colchicus*	Common pheasant	Omnivorous	1	**1 (100%)**
	Alectoris rufa	Red-legged partridge	Grain eater	5	0
	Coturnix coturnix	Common quail	Omnivorous	2	0
Otidiformes	*Tetrax tetrax*	Little bustard	Omnivorous	1	**1 (100%)**
Passeriformes	*Sturnus vulgaris*	Common starling	Insectivorous	22	**5 (22.7%)**
	Turdus philomelos	Song thrush	Omnivorous	10	**4 (40%)**
	Passer domesticus	House sparrow	Omnivorous, Opportunistic	19	**3 (15.8%)**
	Coeloeus monedula	Eurasian jackdaw	Omnivorous, Opportunistic	16	**2 (12.5%)**

	Fringilla coelobs	Common chaffinch	Grain eater, Other	6	**2 (33.3%)**
	Phoenicurus ochruros	Black redstart	Insectivorous	4	**2 (50%)**
	Pica pica	Eurasian magpie	Omnivorous	24	**2 (8.3%)**
	Sylvia melanocephala	Sardinian warbler	Insectivorous, fruit eater	3	**2 (66.6%)**
	Carduelis carduelis	European goldfinch	Grain eater	4	**1 (25%)**
	Spinus spinus	Eurasian siskin	Grain eater	2	**1 (50%)**
	Sylvia atricapilla	Eurasian blackcap	Insectivorous, fruit eater	5	**1 (20%)**
	Troglodytes troglodytes	Eurasian wren	Insectivorous	1	**1 (100%)**
	Turdus merula	Common blackbird	Insectivorous, fruit eater	2	**1 (50%)**
	Chloris chloris	European greenfinch	Grain eater, fruit eater	2	0
	Coccothraustes coccothraustes	Hawfinch	Grain eater, fruit eater	4	0
	Corvus corone	Carrion crow	Omnivorous	1	0
	Cyaniste caeruleus	Eurasian blue tit	Insectivorous	2	0
	Delichon urbica	Common House martin	Insectivorous	4	0
	Emberiza cirlus	Cirl bunting	Grain eater	1	0
	Erithacus rubecula	European robin	Insectivorous	3	0

	Ficedula hypoleuca	European pied flycatcher	Insectivorous	2	0
	Garrulus glandarius	Eurasian jay	Omnivorous	1	0
	Hirundo rustica	Barn swallow	Insectivorous	1	0
	Loxia curvirostra	Red crossbill	Other	1	0
	Parus major	Eurasian great tit	Omnivorous	3	0
	Passer monatus	Tree sparrow	Omnivorous	1	0
	Petronia petronia	Rock sparrow	Grain eater, fruit eater	1	0
	Phoenicurus phoenicurus	Common redstart	Insectivorous	1	0
	Phylloscopus collybita	Common chiffchaff	Insectivorous	1	0
	Phylloscopus trochilus	Willow warbler	Insectivorous	1	0
	Serinus serinus	European serin	Grain eater	2	0
	Sylvia cantillans	Subalpine warbler	Insectivorous, fruit eater	1	0
	Sylvia undata	Dartford warbler	Insectivorous	1	0
	Taeniopygia guttata	Zebra finch	Grain eater, Insectivorous	1	0
Pelecaniformes	*Ardea cinerea*	Grey heron	Other	5	**3 (60%)**
	Bulbulcus ibis	Cattle egret	Insectivorous	2	0

Phoenicopteriformes	*Phoenicopterus roseus*	Greater flamingo	Other	6	**1 (16.6%)**
Podicipediformes	*Podiceps cristasus*	Great crested grebe	Other	2	0
Strigiformes	*Otus scops*	European scops owl	Small vertebrates eater	6	**1 (16.6%)**
	Tyto alba	Barn owl	Small vertebrates eater	3	**1 (33.3%)**
	Aegolius funereus	Boreal owl	Small vertebrates eater	1	0
	Asio flammeus	Short-eared owl	Small vertebrates eater	1	0
	Asio otus	Long-eared owl	Small vertebrates eater	2	0
	Athene noctua	Little owl	Small vertebrates eater	2	0
	Bubo bubo	Eurasian eagle-owl	Small vertebrates eater	4	0
	Strix aluco	Tawny owl	Small vertebrates eater	14	0
Suliformes	*Phalacrocorax carbo*	Great cormoran	Other	3	**1 (33.3%)**
	Morus basanus	Gannet	Other	3	0

Supplementary Data S2: Complete list of birds Orders and species sampled during the study and number of individuals positive to rare *Cryptococcus* and former *Cryptococcus* (RCFC) within each specie

Meaning of main diet column categories :
Grain eaters indicates birds feeding mainly on grain and/or seeds at adult stage;
Fruit eaters indicates birds feeding mainly on fruit and/or berries at adult stage;
Small vertebrate eaters indicates birds feeding on a variety of small terrestrial vertebrate preys such as mice, voles, small reptiles and small birds at adult stage;

Insectivorous indicates birds feeding on a wide variety of insects and other terrestrial arthropods such as spiders at adult stage;
Omnivorous indicates birds with no preferential diet between plants/grain and animals;
Opportunistic indicates birds with a wide variety of food sources that also scavenge on human wastes;
Other group indicates birds with a diet that does not fit into any of the previous groups. This includes specialist feeders, birds feeding on invertebrates other than insects such as annelids, molluscs or crustaceans, birds feeding on aquatic food sources such as fishes or amphibians, carrion eaters….
