# Supplementary material for: Search for Cryptococcus neoformans/gattii Complexes and Related Genera (Filobasidium, Holtermanniella, Naganishia, Papiliotrema, Solicoccozyma, Vishniacozyma) spp. Biotope: Two Years Surveillance of Wild Avian Fauna in Southern France
Source: J Fungi (Basel). 2022 Feb 24;8(3):227. doi: 10.3390/jof8030227 (PMC8948691; doi:10.3390/jof8030227)
Supplement: Supplementary file 1 [file jof-08-00227-s001.zip › Supplementary Data S3.pdf]

**Order**	**Animal sampled**	**Stage**	**Clinical datas**	**Method of sampling**	**Cryptococcal species isolated (actual name)**	**Cryptococcal species isolated (ancient name)**	**Primers used for sequencing**
Accipitriforme	*Accipiter nisus*	**A**	Amoxicillin + clavulanate + meloxicam	Swab on cage wet droppings	*Filobasidium magnum*	*Cryptococcus magnus*	ITS
	Aegypius monachus	**A**	ND	Swab on cage dry droppings	*Naganishia albida*	*Cryptococcus kuetzingii*	NL
	Buteo buteo	**A**	ND	Direct cloacal swab	*Filobasidium wieringae*	*Cryptococcus wieringae*	ITS
	Buteo buteo	**A**	Wounded by lead pellets	Direct cloacal swab	*Naganishia albida*	*Cryptococcus albidus*	NL
	Buteo buteo	**A**	ND	Swab on cage dry droppings	*Filobasidium oeirense*	*Cryptococcus oeirensis*	ITS
	Buteo buteo	**A**	ND	Swab on cage dry droppings	*Naganishia uzbekistanensis*	*Cryptococcus uzbekistanensis*	ITS
	Circaetus gallicus	**A**	Wounded by lead pellets, amoxicillin + meloxicam	Swab on cage wet droppings	*Naganishia diffluens*	*Cryptococcus diffluens*	ITS
	Pernis apivorus	**A**	Wounded by lead pellets	Direct cloacal swab	*Vishniacozyma carnescens*	*Cryptococcus carnescens*	ITS
Anseriforme	*Cygnus olor*	**J**	Botulism, activated coal + rehydratation	Swab on cage wet droppings	*Vishniacozyma carnescens*	*Cryptococcus carnescens*	ITS
Apodiforme	*Apus apus*	**A**	ND	Swab on cage dry droppings	*Cutaneotrichosporon curvatus* + *Naganishia diffluens*	*Cryptococcus curvatus* + *C. diffluens*	ITS<br>ITS

	Apus apus	**A**	ND	Swab on cage wet droppings	*Filobasidium magnum*	*Cryptococcus magnus*	ITS
	Apus apus	**A**	ND	Swab on cage wet droppings	*Filobasidium magnum*	*Cryptococcus magnus*	ITS
Bucerotiforme	*Upupa epops*	**A**	ND	Direct cloacal swab	*Naganishia diffluens*	*Cryptococcus diffluens*	ITS
Charadriiforme	*Chroicocephalus ridibundus*	**J**	Coccidiosis	Swab on cage wet droppings	*Filobasidium floriforme*	*Cryptococcus albidus*	NL
	Larus michaelis	**A**	ND	Direct cloacal swab	*Filobasidium magnum*	*Cryptococcus magnus*	ITS
	Larus michaelis	**J**	ND	Direct cloacal swab	*Naganishia randhawae*	*Cryptococcus randhawai*	ITS
	Larus michaelis	**A**	ND	Swab on cage dry droppings	*Naganishia globosa*	*Cryptococcus saitoi*	ITS
	Larus michaelis	**A**	ND	Swab on cage dry droppings	*Naganishia uzbekistanensis*	*Cryptococcus uzbekistanensis*	ITS
	Larus michaelis	**A**	ND	Swab on cage wet droppings	*Cryptococcus ovalis*	*Cryptococcus ovalis*	ITS
	Larus michaelis	**A**	ND	Swab on cage wet droppings	*Cutaneotrichosporon curvatus*	*Cryptococcus curvatus*	ITS
	Larus michaelis	**A**	Thiamine-Pyridoxine + Vincamine-papaverine + Rapidexon	Swab on cage wet droppings	*Filobasidium magnum*	*Cryptococcus magnus*	ITS
	Larus michaelis	**I**	ND	Swab on cage wet droppings	*Filobasidium magnum*	*Cryptococcus magnus*	ITS

	Larus michaelis	**A**	ND	Swab on cage wet droppings	*Naganishia albida*	*Cryptococcus albidus*	NL
	Larus michaelis	**A**	Avian pox	Swab on cage wet droppings	*Naganishia diffluens*	*Cryptococcus diffluens*	ITS
	Larus michaelis	**A**	ND	Swab on cage wet droppings	*Papiliotrema laurentii*	*Cryptococcus laurentii*	ITS
	Larus michaelis	**A**	ND	Swab on cage wet droppings	*Vishniacozyma carnescens*	*Cryptococcus carnescens*	ITS
	Larus michaelis	**A**	Amoxicillin + clavulanate	Swab on cage wet droppings	*Vishniacozyma carnescens*	*Cryptococcus carnescens*	ITS
	Scolopax rusicola	**A**	ND	Direct cloacal swab	*Naganishia uzbekistanensis*	*Cryptococcus uzbekistanensis*	ITS
Ciconiforme	*Ciconia ciconia*	**A**	Electrocution	Direct cloacal swab	*Naganishia liquefasciens*	*Cryptococcus liquefasciens*	ITS
Columbiforme	*Columba livia*	**A**	Wounded by lead pellets	Direct cloacal swab	*Cryptococcus ovalis*	*Cryptococcus ovalis*	ITS
	Columba livia	**A**	ND	Swab on cage wet droppings	*Filobasidium magnum*	*Cryptococcus magnus*	ITS
	Columba livia	**A**	ND	Swab on cage wet droppings	*Naganishia albida*	*Cryptococcus kuetzingii*	NL
	Columba livia	**J**	ND	Swab on cage wet droppings	*Naganishia uzbekistanensis*	*Cryptococcus uzbekistanensis*	ITS
	Columba palumbus	**A**	ND	Swab on cage dry droppings	*Filobasidium magnum*	*Cryptococcus magnus*	ITS

	Columba palumbus	**A**	ND	Swab on cage dry droppings	*Papiliotrema laurentii*	*Cryptococcus laurentii*	ITS
	Columba palumbus	**A**	ND	Swab on cage wet droppings	*Filobasidium magnum*	*Cryptococcus magnus*	ITS
	Columba palumbus	**A**	ND	Swab on cage wet droppings	*Filobasidium magnum*	*Cryptococcus magnus*	ITS
	Columba palumbus	**A**	ND	Swab on cage wet droppings	*Filobasidium magnum*	*Cryptococcus magnus*	ITS
	Columba palumbus	**A**	ND	Swab on cage wet droppings	*Filobasidium magnum*	*Cryptococcus magnus*	ITS
	Columba palumbus	**J**	ND	Swab on cage wet droppings	*Naganishia adeliensis*	*Cryptococcus adeliensis*	ITS
	Columba palumbus	**A**	ND	Swab on cage wet droppings	*Naganishia diffluens*	*Cryptococcus diffluens*	ITS
	Columba palumbus	**J**	Possible Newcastle disease	Swab on cage wet droppings	*Vishniacozyma carnescens*	*Cryptococcus carnescens*	ITS
	Streptopelia decaocto	**A**	ND	Direct cloacal swab	*Filobasidium magnum*	*Cryptococcus magnus*	ITS
	Streptopelia decaocto	**A**	ND	Direct cloacal swab	*Solicoccozyma aeria + Vishniacozyma carnescens*	*Cryptococcus aerius + C. carnescens*	ITS
	Streptopelia decaocto	**A**	ND	Swab on cage dry droppings	*Filobasidium floriforme*	*Cryptococcus albidus*	NL

	Streptopelia decaocto	**A**	Rapidexon+ meloxicam+etamsylate	Swab on cage dry droppings	*Naganishia albida*	*Cryptococcus albidus*	NL
	Streptopelia decaocto	**A**	ND	Swab on cage dry droppings	*Naganishia albida*	*Cryptococcus albidus*	NL
	Streptopelia decaocto	**A**	ND	Swab on cage dry droppings	*Naganishia albida*	*Cryptococcus albidus*	NL
	Streptopelia decaocto	**A**	ND	Swab on cage wet droppings	*Cryptococcus sp +* *Filobasidium magnum*	*Cryptococcus sp* *+ C.magnus*	ITS and NL + ITS
	Streptopelia decaocto	**A**	Possible Newcastle disease	Swab on cage wet droppings	*Filobasidium floriforme*	*Cryptococcus albidus*	NL
	Streptopelia decaocto	**A**	ND	Swab on cage wet droppings	*Filobasidium magnum*	*Cryptococcus magnus*	ITS
	Streptopelia decaocto	**A**	ND	Swab on cage wet droppings	*Filobasidium magnum*	*Cryptococcus magnus*	ITS
	Streptopelia decaocto	**I**	ND	Swab on cage wet droppings	*Filobasidium magnum*	*Cryptococcus magnus*	ITS
	Streptopelia decaocto	**A**	ND	Swab on cage wet droppings	*Filobasidium magnum*	*Cryptococcus magnus*	ITS
	Streptopelia decaocto	**I**	ND	Swab on cage wet droppings	*Filobasidium magnum*	*Cryptococcus magnus*	ITS
	Streptopelia decaocto	**A**	ND	Swab on cage wet droppings	*Filobasidium magnum*	*Cryptococcus magnus*	ITS

	Streptopelia decaocto	**A**	Amoxicillin + clavulanate + meloxicam	Swab on cage wet droppings	*Filobasidium magnum + Naganishia albida*	*Cryptococcus magnus + C. albidus*	ITS<br>NL
	Streptopelia decaocto	**A**	ND	Swab on cage wet droppings	*Naganishia albida + Papiliotrema flavescens*	*Cryptococcus albidus + C. flavescens*	NL<br>NL
	Streptopelia decaocto	**A**	ND	Swab on cage wet droppings	*Naganishia diffluens*	*Cryptococcus diffluens*	ITS
	Streptopelia decaocto	**A**	ND	Swab on cage wet droppings	*Naganishia globosa*	*Cryptococcus saitoi*	ITS
	Streptopelia decaocto	**A**	ND	Swab on cage wet droppings	*Naganishia globosa*	*Cryptococcus saitoi*	ITS
	Streptopelia decaocto	**A**	ND	Swab on cage wet droppings	*Naganishia uzbekistanensis*	*Cryptococcus uzbekistanensis*	ITS
Falconiforme	*Falco naumanni*	**A**	Meloxicam	Direct cloacal swab	*Naganishia albida*	*Cryptococcus albidus*	NL
	Falco naumanni	**A**	Meloxicam	Swab on cage wet droppings	*Filobasidium magnum*	*Cryptococcus magnus*	ITS
	Falco tinnunculus	**I**	ND	Direct cloacal swab	*Filobasidium oeirense*	*Cryptococcus oeirensis*	ITS
	Falco tinnunculus	**A**	ND	Swab on cage dry droppings	*Filobasidium magnum*	*Cryptococcus magnus*	ITS
	Falco tinnunculus	**A**	ND	Swab on cage dry droppings	*Naganishia albida*	*Cryptococcus kuetzingii*	NL

	Falco tinnunculus	**A**	Meloxicam	Swab on cage dry droppings	*Naganishia diffluens*	*Cryptococcus diffluens*	ITS
	Falco tinnunculus	**A**	Amoxicillin + clavulanate + meloxicam	Swab on cage dry droppings	*Naganishia diffluens* + *Cutaneotrichosporon curvatus*	*Cryptococcus diffluens* + *Cryptococcus curvatus*	ITS<br>ITS
	Falco tinnunculus	**A**	ND	Swab on cage dry droppings	*Naganishia uzbekistanensis*	*Cryptococcus uzbekistanensis*	ITS
	Falco tinnunculus	**A**	ND	Swab on cage wet droppings	*Naganishia albida*	*Cryptococcus albidus*	NL
Galliforme	*Phasianus colchicus*	**A**	ND	Swab on cage dry droppings	*Filobasidium magnum*	*Cryptococcus magnus*	ITS
Otidiforme	*Tetrax tetrax*	**A**	Wounded by lead pellets	Swab on cage wet droppings	*Naganishia globosa*	*Cryptococcus saitoi*	ITS
Passeriforme	*Carduelis carduelis*	**A**	ND	Swab on cage wet droppings	*Holtermaniella festucosa*	*Cryptococcus festucosus*	ITS
	Coloeus monedula	**A**	ND	Swab on cage wet droppings	*Naganishia diffluens*	*Cryptococcus diffluens*	ITS
	Coloeus monedula	**A**	ND	Swab on cage wet droppings	*Vishniacozyma carnescens* + *Naganishia uzbekistanensis*	*Cryptococcus carnescens* + *C. uzbekistanensis*	ITS<br>ITS
	Fringilla coelebs	**A**	Rapidexon	Swab on cage dry droppings	*Filobasidium magnum*	*Cryptococcus magnus*	ITS
	Fringilla coelebs	**A**	ND	Swab on cage wet droppings	*Filobasidium magnum*	*Cryptococcus magnus*	ITS

	Passer domesticus	**A**	ND	Swab on cage dry droppings	*Filobasidium magnum*	*Cryptococcus magnus*	ITS
	Passer domesticus	**A**	ND	Swab on cage dry droppings	*Filobasidium magnum*	*Cryptococcus magnus*	ITS
	Passer domesticus	**A**	ND	Swab on cage wet droppings	*Naganishia liquefasciens*	*Cryptococcus liquefasciens*	ITS
	Phoenicurus ochruros	**J**	ND	Direct cloacal swab	*Filobasidium chernovii*	*Cryptococcus chernovii*	ITS
	Phoenicurus ochruros	**A**	Meloxicam	Swab on cage wet droppings	*Filobasidium magnum*	*Cryptococcus magnus*	ITS
	Pica pica	**A**	ND	Swab on cage dry droppings	*Naganishia albida*	*Cryptococcus kuetzingii*	NL
	Pica pica	**J**	ND	Swab on cage wet droppings	*Naganishia diffluens*	*Cryptococcus diffluens*	ITS
	Spinus spinus	**A**	ND	Swab on cage wet droppings	*Filobasidium magnum*	*Cryptococcus magnus*	ITS
	Sturnus vulgaris	**A**	ND	Direct cloacal swab	*Filobasidium magnum*	*Cryptococcus magnus*	ITS
	Sturnus vulgaris	**A**	ND	Swab on cage wet droppings	*Filobasidium magnum*	*Cryptococcus magnus*	ITS
	Sturnus vulgaris	**A**	ND	Swab on cage wet droppings	*Filobasidium magnum*	*Cryptococcus magnus*	ITS
	Sturnus vulgaris	**A**	ND	Swab on cage wet droppings	*Papiliotrema flavescens*	*Cryptococcus flavescens*	ITS

	Sturnus vulgaris	**A**	ND	Swab on cage wet droppings	*Papiliotrema laurentii*	*Cryptococcus laurentii*	ITS
	Sylvia atracapilla	**A**	ND	Swab on cage dry droppings	*Naganishia globosa*	*Cryptococcus saitoi*	ITS
	Sylvia melanocephala	**A**	ND	Direct cloacal swab	*Papiliotrema flavescens + N. albida*	*Cryptococcus flavescens + C. albidus*	ITS<br>NL
	Sylvia melanocephala	**A**	ND	Swab on cage dry droppings	*Naganishia albida*	*Cryptococcus albidus*	NL
	Troglodytes troglodytes	**I**	ND	Swab on cage wet droppings	*Vishniacozyma carnescens*	*Cryptococcus carnescens*	ITS
	Turdus merula	**A**	ND	Swab on cage wet droppings	*Papiliotrema terrestris*	*Cryptococcus terrestris*	ITS
	Turdus philomelos	**A**	ND	Direct cloacal swab	*Solicoccozyma aeria*	*Cryptococcus aerius*	ITS
	Turdus philomelos	**A**	ND	Swab on cage dry droppings	*Filobasidium magnum*	*Cryptococcus magnus*	ITS
	Turdus philomelos	**A**	ND	Swab on cage dry droppings	*Filobasidium magnum + Naganishia albida*	*Cryptococcus magnus + C. albidus*	ITS<br>NL
	Turdus philomelos	**A**	ND	Swab on cage wet droppings	*Papiliotrema terrestris*	*Cryptococcus terrestris*	ITS

Pelecaniforme	*Ardea cinerea*	**A**	ND	Swab on cage dry droppings	*Vishniacozyma carnescens*	*Cryptococcus carnescens*	ITS
	Ardea cinerea	**A**	ND	Swab on cage wet droppings	*Naganishia diffluens*	*Cryptococcus diffluens*	ITS
	Ardea cinerea	**A**	ND	Swab on cage wet droppings	*Naganishia uzbekistanensis*	*Cryptococcus uzbekistanensis*	ITS
Phoenicoptiforme	*Phoenicopterus roseus*	**A**	ND	Swab on cage wet droppings	*Filobasidium chernovii*	*Cryptococcus chernovii*	ITS
Strigiforme	*Tyto alba*	**A**	ND	Direct cloacal swab	*Filobasidium floriforme*	*Cryptococcus albidus*	NL
	Otus scop	**A**	ND	Swab on cage dry droppings	*Naganishia diffluens*	*Cryptococcus diffluens*	ITS
Suliforme	*Phalacrocorax carbo*	**A**	Amoxicillin + clavulanate + meloxicam	Swab on cage wet droppings	*Filobasidium magnum*	*Cryptococcus magnus*	ITS

Supplementary Data S3: Detailed Distribution of rare *Cryptococcus* and former *Cryptococcus* species by bird of origin and method of sampling

A : indicates an Adult; ; J : indicates a Juvenile; I : indicates an Immature bird. ND indicates no specific clinical data. ITS indicates that sequences were obtained by ITS1-ITS4 sequencing. NL indicates that sequences were obtained by NL1-NL4 sequencing.
