# Supplementary figures and images for: Search for Cryptococcus neoformans/gattii Complexes and Related Genera (Filobasidium, Holtermanniella, Naganishia, Papiliotrema, Solicoccozyma, Vishniacozyma) spp. Biotope: Two Years Surveillance of Wild Avian Fauna in Southern France
Source: J Fungi (Basel). 2022 Feb 24;8(3):227. doi: 10.3390/jof8030227 (PMC8948691; doi:10.3390/jof8030227)

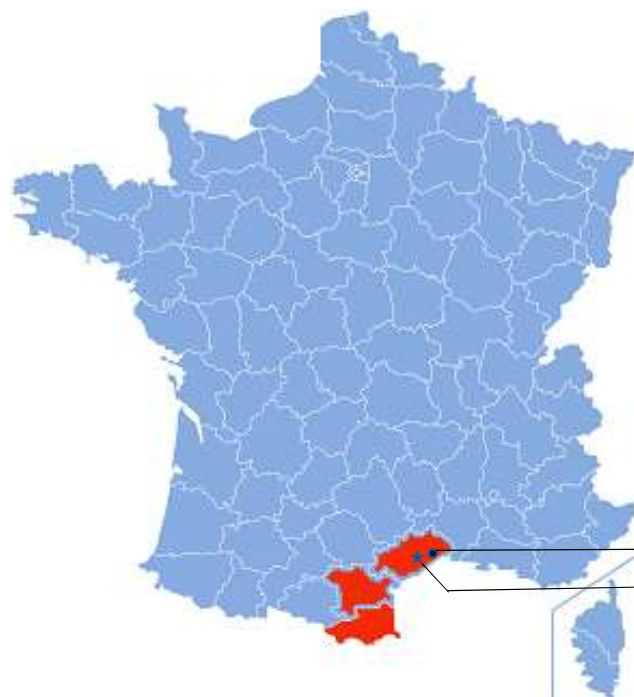

Montpellier 43° 36' 38.768" N 3° 52' 36.178" E  
Centre de Sauvegarde de la Faune Sauvage  
43°28'47.6"N 3°36'49.3"E

Supplement: Supplementary file 1 [file jof-08-00227-s001.zip › Supplementary Data S1.pdf]
